# “Diminishing returns” and leaf area-biomass scaling of ferns in subtropical ecosystems

**DOI:** 10.3389/fpls.2023.1187704

**Published:** 2023-06-27

**Authors:** Shubing Chen, Jinlong Li, Jun Sun, Quanlin Zhong, Dandan Hu, Dongliang Cheng

**Affiliations:** ^1^ College of Geographical Sciences, Fujian Normal University, Fuzhou, China; ^2^ Fujian Provincial Key Laboratory of Plant Ecophysiology, Fujian Normal University, Fuzhou, China; ^3^ Key Laboratory of Humid Subtropical Eco-Geographical Process, Ministry of Education, Fuzhou, China; ^4^ School of Resources and Environment, Anqing Normal University, Anqing, China

**Keywords:** leaf area-biomass relationship, diminishing returns hypothesis, elevation, subtropical ecosystem, ferns

## Abstract

Foliage leaves are the primary photosynthetic organ of the majority of vascular plants, and their area vs. biomass scaling relationships provide valuable insights into the capacity and investment in light interception, which is critical to plant growth and performance. The “diminishing returns” hypothesis (DRH), which is based primarily on data from gymnosperms and angiosperms, posits that leaf (lamina) area scales with leaf dry mass. on average with a scaling exponent less than 1.0. However, it remains uncertain whether DRH applies to ferns or whether ecological factors affect the scaling exponents governing fern leaf morphometrics. To address this issue, 182 individuals of 28 subtropical ferns species were studied at low, medium, and high elevations (i.e., 600 m, 900 m, and 1200 m, respectively) in Mount Wuyi National Park, Jiangxi Province, China. The scaling relationships between leaf area and leaf biomass for individual and total leaf of ferns at different elevations were examined by using standardized major axis regression protocols. Analyses of the 28 fern species (using Blomberg *K*-value protocols) indicated no phylogenetic biases among the species compositions of the three different elevations. In addition, at the individual plant level, individual leaf area (ILA) did not differ significantly among the three different elevations (*P* > 0.05). However, individual leaf mass (ILM) was significantly higher at 900m than at 1200m (*P* < 0.05), resulting in a significantly higher leaf mass per area (LMA) at the 900m elevation than at the 600m and 1200m elevations (*P* < 0.05). The ILA and ILM at the 900m elevation were significantly higher than at the 600m elevation (*P* < 0.05). At the species level, ILA and ILM did not differ significantly among the three elevations (*P* > 0.05). The total leaf area per individual (TLA) did not differ significantly across the different elevations (*P* > 0.05). However, total leaf mass per individual (TLM) did differ significantly (*P* < 0.05). At the individual plant level, the scaling exponents for ILA vs. ILM and TLA vs. TLM at the three different elevations were all significantly less than 1.0 (*P* < 0.05), which was consistent with the DRH. At the species level, the scaling exponents for the ILA vs. ILM were significantly less than 1.0 at the middle and high elevations, but not at the low elevation. The scaling exponents of the TLA and TLM were numerically highest in the middle elevation, and all were less than 1.0 for the three elevations. These results indicate that the scaling relationships of leaf area versus mass of subtropical ferns at different elevations support the DRH hypothesis. The study further informs our understanding of the resource allocation strategies of an ancient and diverse plant lineage.

## 1 Introduction

The relationship between leaf (lamina) biomass and area not only reflects the light-harvesting capability of most land plants, but also their environmental adaptability (Kleiman and Aarssen, 2007; Huang et al., 2020; Guo et al., 2022). Therefore, this scaling relationship has important implications for plant growth and community productivity (Liu et al., 2020).

Typically, dry leaf biomass (*M*_L_) characterizes the cost of leaf construction, whereas leaf area (*A*_L_) characterizes the potential for photosynthetic gain. Prior work has shown that *A*_L_ is statistically significantly related to *M*_L_ and takes the form of a power function, i.e., *A*_L_ =β *M*_L_^α^, where α is the scaling exponent and β is the scaling (normalization) constant. When α < 1.0, leaf area increases more slowly relative to increasing leaf biomass, a phenomenon called “diminishing returns”. When α > 1.0, there is a gain in leaf area relative to increasing leaf biomass. An isometric (“break even”) relationship occurs when α = 1.0 (Niklas et al., 2007). By determining the leaf area vs. leaf biomass scaling relationships of six functional species groups and 19 individual species, Niklas et al. (2007) confirmed the “diminishing returns” hypothesis (DRH) wherein α < 1.0. Other studies have explored the DRH for plants at different geographic scales and different regions (Shipley, 1995; Pearcy et al., 2005). For example, Shi et al. (2019) showed that leaves area vs. dry mass scaling relationship were in accordance with the DRH in all examined vines leaves. Sun et al. (2017) showed that all five species of bamboo leaves are consistent with the DRH. More importantly, the total leaf area-biomass of whole plant should follow the DRH as the individual leaf area- biomass. Indeed, Zhu et al. (2019) found that DRH applied to the scaling relationship between total leaf area and total leaf biomass in current twigs of 64 subtropical woody species.

However, plant growth and development are affected by the environment (Zhang et al., 2020), and plant biomass exhibits the same or different scaling relationship at different elevations likely in response to hydrothermal conditions (Korner et al., 2001). Thus, it is reasonable to surmise that the scaling of leaf area with respect to leaf biomass may differ across gradients of temperature, water availability, and elevation. Yang et al. (2012) found that leaf size is smaller in species adapted to high elevations compared to species adapted to lower elevations, and report that an elevation gradient can significantly affect the trade-off between leaf area and leaf number. However, Sun et al. (2017) showed that the scaling exponents for the leaf area vs. dry mass scaling relationship are less than 1.0 within and across five species of bamboo and are insensitive to light conditions and elevation. Geng et al. (2019) found that the slope of leaf area and leaf biomass of *Disanthus cercidifolius* was significantly different from 1.0 at low altitudes, whereas at mid and high altitudes the slope was not significantly different from 1.0, i.e., an isotropic relationship. These different findings indicate that differences in elevation can have an effect on the scaling relationship of plants under different circumstances.

Importantly, the DRH is based primarily on data drawn from gymnosperms and angiosperms. Consequently, little is known about the scaling of non-seed plant species, such as ferns, particularly those growing in tropical forest ecosystems. Ferns represent a unique and extremely ancient evolutionary group of vascular plants (Bierhorst, 1971; Page, 2002) and have an important position and role in the structure, function, and dynamics of forest ecosystems (Li et al., 2000). The response of ferns to external natural conditions is highly sensitive and selective (Schneider et al., 2004; Jin et al., 2021), significantly influencing the dynamic processes of forest communities, and can be important for the occurrence and succession of communities, nutrient cycling, and energy flow (Yan et al., 2004). However, as noted, most current studies on plant functional traits have been focused on gymnosperm and angiosperms species with self-supporting stems (Westoby et al., 2002; Shipley et al., 2006). Far fewer studies have dwelt on the relationship between leaf area and leaf biomass in rhizomatous species. Fern fronds are often the only above-ground part of the plant body and are therefore the primary photosynthetic organs (Mao et al., 2012). Therefore, studying the relationship between frond area and biomass and its adaptation to different environments can inform our understanding of the adaptation strategies of an ancient and diverse lineage growing under different conditions.

The subtropical region of China has high forest cover, soil fertility, and biodiversity. It therefore provides a rich natural environmental context to study fern biology. The typical middle subtropical forest ecosystem of Wuyi Mountain was selected for study because of its huge variety of ferns that are widely distributed. In this study, ferns at different elevations were selected to determine the leaf area vs. biomass scaling relationship and to determine whether there are differences in the numerical values of the scaling exponents of the leaf area vs. leaf biomass scaling relationship at different elevations.

## 2 Materials and methods

### 2.1 Study area

The study area is located in the Wuyi Mountain National Park in southeastern of China. The area has a subtropical monsoon climate, and is the largest and most completely preserved meso-subtropical forest ecosystem in the world. The average annual precipitation is 1486-2150 mm and is concentrated in April-June. The average annual temperature is 8.5-18°C. The highest peak in the reserve is Huanggang Mountain. As the elevation decreases, the soil vertical zones are mountain meadow soil zone, yellow soil zone, yellow-red soil zone, and red soil zone (Chen, 2000).

### 2.2 Sample collection and index determination

Twenty-eight plants with healthy and fully mature leaves were selected for each species at elevations of 600 m, 900 m, and 1200 m, and we verified on website (http://www.sp2000.org.cn/) that all fern species included in the sample were native. The individual plants were excavated and placed into coded sealed bags and then brought back to the laboratory. Leaves were removed from rhizomes and their lamina were scanned using a scanner (EPSON V39, Japan). The scanned images were processed using ImageJ software to obtain leaf area data. The scanned leaf samples were then dried in an oven at 75°C for 48 hours to constant weight and weighed using an electronic scale with an accuracy of 0.001 g. LMA was calculated as the leaf dry weight per leaf area (mg/mm²). The leaf (lamina) area and dry weight were measured and calculated the leaf (lamina) area and dry weight for each fern samples collected. The ILM at individual plant level was calculated as the total leaf mass of each sample divided by the number of leaves. Similarly, the total leaf mass per individual (TLM) was calculated as the sum of the leaf biomass of each fern plant harvested. At the species level, the ILM was calculated as the average value of the TLM of the sample divided by the number of leaves. The TLM was calculated as the average of the sum of the total leaf biomass of each fern harvested at the species level. Similarly, the individual leaf area (ILA) and total leaf area per individual (TLA) were calculated in the same way.

## 3 Analysis

The data were processed using Excel 2016 software. The mean and standard errors were calculated for the leaf area and dry weight of individual plants using SPSS 19.0. The differences between leaf area, biomass, and mass per area at different elevations were analyzed using one-way ANOVA.

The *K*-value method proposed by Blomberg et al. (2003) was used to measure the strength of the phylogenetic signal for continuous functional traits. It detects correlations between functional traits and species evolutionary history, and allows comparisons between traits and between phylogenetic trees. If the *K*-value is greater than 1, the functional traits under investigation show a stronger phylogenetic signal than in the Brownian motion model. If the *K*-value is less than 1, the functional traits show a weaker phylogenetic signal than the Brownian motion model. Under Brownian motion, evolutionary changes are simply added to the values that occurred in the previous generation or at the last node on the phylogenetic tree. Consequently, lineages that have recently diverged will exhibit a higher degree of similarity (on average), compared to the lineages that are more distantly related (Blomberg et al., 2003). We built a phylogenetic tree using the “V. PhyloMaker2” package in the R(4.0.3)(Jin and Qian, 2022), and the K values in this study were analyzed in R using the *phylosignal()* function in the package “picante” (Kembel et al., 2010). The data were log_10_-transformed and leaf area and biomass at different elevations were analyzed in R (4.0.3) using the “smatr” package to determine the scaling relationships among the variables of interest, i.e., *A*_L_ =β *M*_L_^α^, where β is the scaling (normalization) constant, α is the scaling exponent, and *A*_L_ and *M*_L_ are leaf area and leaf biomass, respectively. In addition, we assessed the scaling relationships of shared species across three different elevations. To compare the exponent associated with each of the three elevations, we used one-way ANOVA in SPSS 19.0. The analysis was performed at the species level using the mean of each variable of the species. Origin 2019 was used to plot the data.

## 4 Results

### 4.1 Characteristics of fern leaf area and leaf biomass across different elevations

The data indicated that the phylogenetic differences of individual leaf mass (ILM), individual leaf area (ILA), and leaf mass per area (LMA) of the plants from the three elevations of ferns were not significant (**S1**; **Table 1**).

**Table 1:** Phylogenetic signal of traits in subtropical ferns.

Functional traits	*K*	*P*
ILM	0.085	0.844
ILA	0.091	0.842
LMA	0.246	0.520

For a detailed description of K statistics see Blomberg et al. (2003).

In addition, the numerical values of the scaling exponents of two fern species (*Phegopteris decursive-pinnata* and *Dicranopteris pedata*) growing at each of the three elevations were used to assess whether exponents at the species level were affected by elevation (**Table 2**). The scaling exponents of ILA and ILM for *P. decursive-pinnata* were 0.82, 0.60, 0.78 at 600m, 900m, and 1200m, respectively (**Table 2**), and the respective scaling exponents for *D. dichotoma* were 1.60, 0.90, 0.88 at the three elevations (**Table 2**). Based on their 95% Cis, these results indicated that the values of the scaling exponents of these two species were, by in large, insensitive to elevation, although one of the scaling relationships was statistically insignificant (e.g., *D. dichotoma* at 600 m; **Table 2**).

**Table 2:** (S)MATR reduced major axis regression slopes and *y*-intercepts (α and log β, respectively) for log_10_-tranformed data for individual leaf area and leaf biomass for *Phegopteris decursive-pinnata* and *Dicranopteris pedata* across different elevations.

Species	Elevation(m)	α (95% CIs)	Log β	*P*	*r*²
*Phegopteris decursive-pinnata*	600	0.82 (0.57,1.16)	2.49	< 0.05	0.96
*Dicranopteris pedata*		1.60 (0.47,5.46)	0.59	> 0.05	0.28
*Phegopteris decursive-pinnata*	900	0.60 (0.45,0.81)	2.35	< 0.05	0.97
*Dicranopteris pedata*		0.90 (0.37,2.18)	2.19	> 0.05	0.89
*Phegopteris decursive-pinnata*	1200	0.78 (0.34,1.77)	2.52	> 0.05	0.75
*Dicranopteris pedata*		0.88 (0.81,0.97)	2.25	< 0.05	0.998

α is the scaling exponent and logβ is the scaling (normalization) constant.

**Figure 1:**
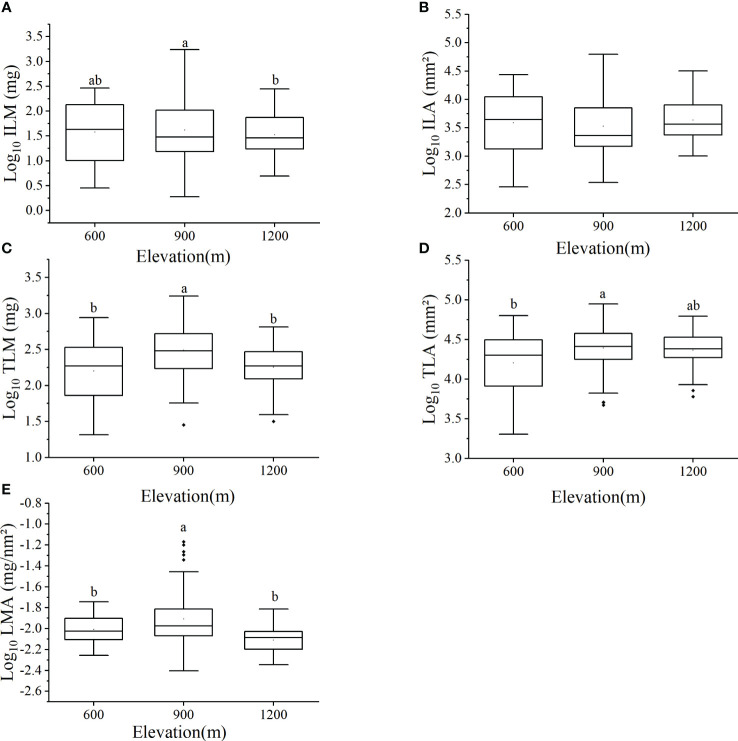
Leaf traits of 28 species of subtropical ferns at different elevations including: **(A)** individual leaf mass, **(B)** individual leaf area, **(C)** total leaf mass per individual, **(D)** total leaf area per individual, **(E)** leaf mass per area. Letters above each plot indicate statistical differences (*P* < 0.05). No letters marked mean no statistical differences [*P* > 0.05, see **(B)**].

The leaf biomass of individual species at different elevations was species-specific and differed but not significantly as a function of elevation (**Table 3**). The mean ILM was 149.75 ± 335.53 mg at the 900m elevation, which was significantly higher than the ILM of 57.41 ± 66.01 mg at the 1200m elevation (**Figure 1A**). However, no statistically significant difference in ILA was detected across elevations (*P* > 0.05, **Figure 1B**), although ILM varied significantly within each elevation. Specifically, the difference between the maximum and minimum values of ILM was 37, 269, and 22 times in three elevations, respectively, whereas LMA varied significantly within each elevation. Specifically, LMA was 15, 11 and 2 times between the maximum and minimum in 600m, 900m and 1200m, respectively (**Table 3**).

**Table 3:** Mean (± SE) number of leaves per plant (*N*), average biomass per leaf, average area per leaf, and average specific leaf weight for each of 38 species of subtropical ferns.

Elevation (m)	Species	*N*	ILM/mg	ILA/mm²	LMA mg/mm²
1200	*Osmunda japonica*	8.60 ± 0.24	22.42 ± 3.13	2876.70 ± 266.10	0.008 ± 0.0004
	*Woodwardia japonica*	4.80 ± 0.37	92.47 ± 3.58	9253.38 ± 748.12	0.010 ± 0.0005
	*Coryphopteris japonica*	8.00 ± 0.32	9.40 ± 1.26	1960.51 ± 268.03	0.005 ± 0.0002
	*Diplopterygium glaucum*	8.00 ± 0.32	18.50 ± 2.87	2207.42 ± 349.33	0.009 ± 0.0007
	*Metathelypteris laxa*	6.00 ± 0.32	27.69 ± 3.43	4660.62 ± 2921.72	0.006 ± 0.0002
	*Dicranopteris pedata*	3.20 ± 0.37	123.88 ± 32.66	12472.13 ± 2921.72	0.010 ± 0.0003
	*Athyrium vidalii*	5.20 ± 0.37	42.94 ± 12.30	4919.77 ± 1066.29	0.008 ± 0.0008
	*Odontosoria chinensis*	7.00 ± 0.32	8.91 ± 2.67	1521.37 ± 428.25	0.006 ± 0.0004
	*Phegopteris decursive-pinnata*	1.40 ± 0.24	199.72 ± 37.69	20126.31 ± 3554.54	0.010 ± 0.0014
	*Pentarhizidium orientale*	6.00 ± 0.41	54.04 ± 5.56	6186.48 ± 771.84	0.009 ± 0.0008
	*Plagiogyria adnata*	7.80 ± 0.37	30.84 ± 7.94	3165.59 ± 547.20	0.009 ± 0.0007
900	*Pyrrosia sheareri*	1.00 ± 0	1351.88 ± 138.48	49579.34 ± 4575.37	0.027 ± 0.0007
	*Cyrtomium fortunei*	13.20 ± 0.86	26.24 ± 3.73	2237.83 ± 285.14	0.012 ± 0.0009
	*Pteris multifida*	9.00 ± 0	14.63 ± 0.82	1964.75 ± 160.18	0.008 ± 0.0003
	*Dryopteris fuscipes*	10.00 ± 0.89	45.57 ± 15.57	3509.13 ± 853.42	0.012 ± 0.0011
	*Parathelypteris glanduligera*	11.60 ± 0.75	12.15 ± 1.38	1458.73 ± 107.50	0.008 ± 0.0006
	*Arachniodes aristata*	6.40 ± 0.68	57.82 ± 10.62	5300.23 ± 1135.44	0.011 ± 0.0007
	*Woodwardia japonica*	5.60 ± 0.40	97.11 ± 10.48	7972.40 ± 1078.11	0.012 ± 0.0005
	*Pyrrosia lingua*	5.00 ± 0.58	224.27 ± 13.01	9333.37 ± 261.72	0.024 ± 0.0007
	*Lepisorus obscurevenulosus*	3.00 ± 0	248.45 ± 44.91	10418.70 ± 1250.86	0.024 ± 0.0030
	*Phegopteris decursive-pinnata*	3.00 ± 0	5.01 ± 1.52	571.21 ± 104.80	0.008 ± 0.0010
	*Microlepia marginata*	18.60 ± 1.03	19.26 ± 5.38	2315.36 ± 564.52	0.008 ± 0.0004
	*Hypolepis punctata*	11.80 ± 1.07	12.69 ± 3.74	2312.43 ± 547.27	0.005 ± 0.0005
	*Dicranopteris pedata*	3.50 ± 0.29	253.78 ± 41.94	22619.06 ± 3113.03	0.011 ± 0.0006
	*Diplopterygium glaucum*	8.00 ± 0.58	40.69 ± 8.25	3947.46 ± 557.00	0.010 ± 0.0001
	*Osmunda japonica*	12.00 ± 0	20.84 ± 4.64	1835.36 ± 150.09	0.011 ± 0.0013
	*Onychium japonicum*	15.40 ± 0.40	16.77 ± 0.78	1567.40 ± 118.16	0.011 ± 0.0011
600	*Lepisorus fortunei*	1.80 ± 0.20	166.27 ± 30.57	13144.31 ± 2116.97	0.013 ± 0.001
	*Diplazium virescens*	6.80 ± 0.66	23.19 ± 6.79	2683.88 ± 689.08	0.008 ± 0.0005
	*Cyrtomium fortunei*	5.80 ± 0.37	34.44 ± 9.32	3464.43 ± 511.72	0.009 ± 0.001
	*Pteris multifida*	4.60 ± 0.75	38.71 ± 8.84	3805.58 ± 1010.45	0.010 ± 0.001
	*Goniophlebium niponicum*	8.00 ± 0.32	5.38 ± 1.01	624.57 ± 73.94	0.008 ± 0.0007
	*Woodwardia prolifera*	3.60 ± 0.24	202.12 ± 33.51	13465.30 ± 1819.83	0.015 ± 0.0009
	*Coniogramme centrochinensis*	3.20 ± 0.49	109.22 ± 17.99	11477.36 ± 1819.83	0.009 ± 0.0007
	*Deparia petersenii*	7.80 ± 0.37	7.21 ± 2.48	841.36 ± 215.60	0.008 ± 0.0008
	*Phegopteris decursive-pinnata*	1.60 ± 0.24	119.76 ± 40.74	14901.89 ± 3811.98	0.008 ± 0.0006
	*Odontosoria chinensis*	6.20 ± 0.37	14.79 ± 7.33	1303.11 ± 548.11	0.010 ± 0.001
	*Dicranopteris pedata*	3.60 ± 0.40	155.57 ± 7.04	12718.07 ± 957.47	0.012 ± 0.0008

The average value of total leaf mass per individual (TLM) at 900m elevation was 428.46 ± 372.10 mg, which was significantly higher than that at 600m elevation (244.10 ± 211.40 mg) and at 1200 elevation (222.71 ± 142.42 mg, *P* < 0.05, **Figure 1C**). Total leaf area per individual (TLA) at 600m elevation (21664.52 ± 15145.01 mm²) was significantly lower than that of 900 elevation (29735.54 ± 17287.02 mm², *P* < 0.05, **Figure 1D**). The LMA of ferns growing at 900m elevation (0.015 ± 0.013 mg/mm²) was significantly higher than that of ferns at 600m elevation (0.010 ± 0.003 mg/mm²) and at 1200m elevation (0.008 ± 0.002 mg/mm²; *P* < 0.05, **Figure 1E**).

### 4.2 The scaling relationship between fern leaf area and leaf biomass at the individual plant level

**Figure 2:**
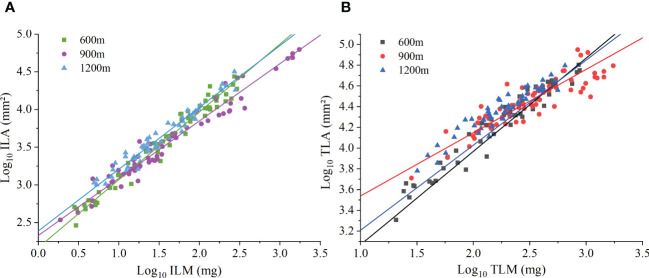
The scaling relationship between leaf area and biomass at the individual level. **(A)** Individual leaf area (ILA) versus individual leaf mass (ILM). **(B)** Total leaf area per individual (TLA) versus total leaf mass per individual (TLM).

At the individual plant level, species manifested a significant ILA vs. ILM scaling relationship at each of the three elevations (**Figure 2A**). The scaling exponents of ILA vs. ILM at 600 m,900 m, and 1200 m were 0.90 (95% CI = 0.85-0.94), 0.76 (95% CI = 0.73-0.80), and 0.83 (95% CI = 0.71-0.97), respectively. All were significantly less than 1.0 (*P* < 0.01), thereby validating the DRH (**Table 4**). TLA vs. TLM scaling relationship was also statistically significant for each of the three elevations. The scaling exponents of TLA vs. TLM at 600m, 900, and 1200m were 0.84 (95% CI = 0.78-0.89), 0.61 (95% CI = 0.55-0.68), and 0.71 (95% CI = 0.65-0.77), respectively. The scaling exponent at the different elevations were significantly less than 1.0 (*P* < 0.01, **Figure 2B**). At the different elevations, the scaling (normalization) constant of individual leaves increased with increasing elevation, while the scaling (normalization) constant at the whole plant level showed a tendency to increase and then decrease with increasing elevation (**Table 4**). These results indicate that the DRH at the level of individual leaves and whole plants leaves remained valid.

**Table 4:** (S)MATR reduced major axis regression slopes and *y*-intercepts (α and logβ, respectively) for log_10_-tranformed data for the individual leaf area vs. leaf biomass scaling relationships of individual plants and at the species level for different elevations.

E(m)	Individual level							Species level						
	N	α(95%CI)		logβ		r²		N	α(95%CI)		logβ		r²	
		Individual leaf	Whole plant	Individual leaf	Whole plant	Individual leaf	Whole plant		Individual leaf	Whole plant	Individual leaf	Whole plant	Individual leaf	Whole plant
600	4.82 ± 0.32	0.90 (0.85,0.90)	0.84 (0.78, 0.89)	2.17	2.37	0.97	0.94	4.82± 0.68	0.92 (0.83, 1.02)	0.86 (0.74, 0.99)	2.12	2.32	0.98	0.96
900	9.32 ± 0.59	0.76 (0.73, 0.80)	0.61 (0.55, 0.68)	2.33	2.93	0.95	0.78	9.17 ± 1.21	0.77 (0.68, 0.86)	0.59 (0.45, 0.77)	2.32	2.97	0.96	0.79
1200	6.00 ± 0.31	0.82 (0.77, 0.86)	0.71 (0.65, 0.77)	2.39	2.80	0.95	0.90	6.00 ± 0.67	0.81 (0.70, 0.93)	0.67 (0.53, 0.84)	2.40	2.86	0.97	0.90

Values are means ( ± SE); E is the elevation of the samples. N is the number of leaves per plant.

### 4.3 The scaling relationship between leaf area and leaf biomass at the species level

**Figure 3:**
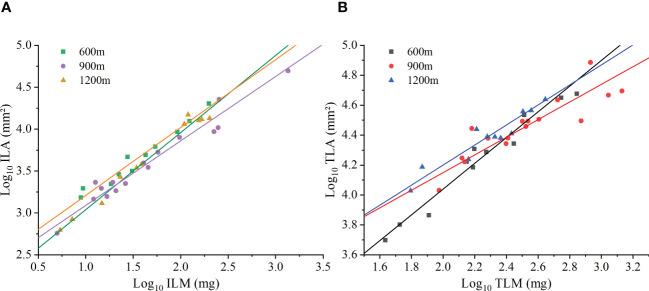
The scaling relationship between leaf area and biomass at the species level. **(A)** Individual leaf area (ILA) versus individual leaf mass (ILM). **(B)** Total leaf area per individual (TLA) versus total leaf mass per individual (TLM).

At the species level, the ferns investigated in this study had an isometric scaling relationship at 600m elevation, but had an allometric ILA vs. ILM scaling relationship at 900m and 1200m elevation **Figure 3A**. The isometric exponent of ILA vs. ILM at 600m elevation was 0.92 (95% CI = 0.83-1.02), which was not significantly different from 1.0 (*P* > 0.05; **Table 4**). The scaling exponent of ILA vs. ILM at 900m and 1200m elevations were 0.77 (95% CI = 0.68-0.87) and 0.81 (95% CI = 0.70-0.93), respectively, both of which were significantly less than 1.0 (*P* < 0.05). In addition, the ILA vs. ILM scaling relationship did not differ significantly among the three different elevations (*P* > 0.05).

At the species level, the scaling exponents of TLA vs. TLM were 0.86 (95% CI = 0.74-0.99), 0.59 (95% CI = 0.45-0.77), and 0.67 (95% CI = 0.53-0.84) for the 600 m, 900 m, and 1200 m, respectively. Each was significantly less than 1.0 (*P* < 0.01, **Figure 3B**; **Table 4**). However, each exponent was statistically different at the three different elevations. The TLM was not significantly different among the three elevations (*P* > 0.05), and LMA were different among elevations (*P* < 0.05), the TLM was significantly different between middle and high elevations (*P* < 0.05). The TLM at 900 elevation (442.66 ± 88.33 mg) was significantly higher than 1200m elevation (117.14 ± 35.32 mg, *P* < 0.05). The ILA (75.23 ± 29.40mm²) was significantly higher in the 900m than that at 600m elevation (71.30 ± 17.76 mm², *P* < 0.05, **Figure 3B**). Likewise, the scaling (normalization) constant of individual leaves increased with increasing elevation, whereas the scaling (normalization) constant at the whole plant level increased and then decreased with increasing elevation (**Table 4**).

## 5 Discussion

The scaling relationship between leaf area and leaf biomass is important for understanding the investment trade-off strategy of plants because it reflects a ‘profit’-’investment’ strategy for photosynthesis and thus plant growth (Niklas et al., 2007). Compared with seed plants, there is a paucity of research on the functional characteristics of fern leaves despite the antiquity of this pteridophyte lineage and its ecological importance in tropical biomass. Therefore, studying the relationship between fern leaf area and leaf biomass at different elevations can further inform our understanding of the trade-off between photosynthetic gain versus biomass investment.

Our study shows that the scaling exponents of leaf area versus leaf biomass are consistently less than 1, this result is consistent with previous studies that there is a significant allometric relationship between the lamina area and mass (Sun et al., 2006; Huang et al., 2019; Jiao et al., 2022). However, our results differ at different elevations at both the individual leaf level and at the species level (**Table 4**). These results support the hypothesis called diminishing returns which predicts that increases in leaf area fail to keep pace with increases in dry biomass investments (Niklas et al., 2007). Additionally, the results indicate that differences in elevation can alter the scaling relationship between individual leaf area (ILA) and biomass (ILM).

### 5.1 Leaf area vs. leaf biomass relationships at the individual level at different elevations

At the whole plant level, the scaling exponent of the leaf area vs. leaf biomass relationship varied numerically at the three different elevations in this study, indicating that ferns have different strategies for leaf biomass investment at different elevations. This finding is consistent with previous research by Sun et al. (2017) on bamboo, which examined the relationship between leaf area and leaf biomass of bamboo at two different elevations in the Wuyi Mountain, southeast China, and found that the scaling exponent of both elevations was less than 1.0 but numerically different. Additionally, our findings are consistent with those of Geng et al. (2019), who discovered that the leaf area and leaf dry biomass scaling relationship of long-stalked bilberry differed with elevation although they confirmed the diminishing returns hypothesis.

However, prior investigation have reported data that contradict the concept of diminishing returns. For example, Pan et al. (2013) analyzed the relationship between leaf area and leaf biomass of 121 vascular plant species and found that the scaling exponents undergo numerical changes from values less than one to greater than one with increasing elevation. These divergent findings suggest that the scaling relationship between leaf area and leaf biomass is influenced by external environment condition and species composition.

### 5.2 Relationship between leaf area vs. leaf biomass at the species level at different elevations

At the species level, the relationship between ILA and ILM is isometric at the low elevation and follows an allometric scaling relationship at the middle and high elevations. We speculate that ferns may invest more biomass at higher elevation to increase the support of the lamina, which in turn may improve the light capture capacity and photosynthetic efficiency of leaves (Ackerly et al., 2002; Ishida et al., 2005; Niinemets et al., 2007). The plasticity of plant morphology in response to environmental variations is an important feature of plant that allows plants to allocate water and nutrients and other resources to the most needed tissues and organs by changing its structure and function, thus maximizing plant competitiveness (Li et al., 2013). It is important to bear in mind that changes in the numerical value of scaling exponents reflect adjustments in the proportional allocations in response to external environmental factors (Shi et al., 2015). The Changes in elevation gradient in mountain areas are influenced by many factors such as changes in light conditions, air pressure, temperature, and humidity, and soils (Chen and Wang, 2021). In this study, Wuyi National Park were characterized by aquatic hydrophytic vegetation at the low elevation, a predominantly bamboo forests at the middle elevation, and tall trees forested community are densely distributed at the high elevation. In the context of this study, it is noteworthy that previous studies suggest that the leaf dry mass per unit leaf (LMA) of plants increased with the increasing light availability (Ackerly et al., 2002). The changes in the numerical values of the leaf area vs. dry mass scaling relationship appear to reflect a greater investment in leaf area at the lowest and highest elevations, and a decreased investment at the mid-elevation. At the lowest and highest elevations, light may be the main primary limiting factor affecting plant access to resources. Therefore, the photosynthetic capacity per unit leaf area decreases under these conditions, making it important to increase the size of leaf area lamina. This adaptive response is significant as larger leaves tend to get more light resources, while small leaves can only intercept light through other parts (petiole, leaf angle) and features (leaf shape) an adaptive response (Ninemets et al., 2004). At the mid-elevation, on the contrary as the elevation increases, the temperature gradually decreases, strong radiation and winds have an impact on the productive performance of plants. In this case the plant leaves then need to allocate more biomass to support structures, the size of the leaf area directly affects the area under stress, so there is a significant difference in the proportion of biomass obtained by large and small leaves, while small leaves have a smaller LMA and thus a greater input to the support tissue.

In this study, leaf area, leaf biomass, and LMA were significantly higher at the mid-altitude than at low and high altitudes. Leaf biomass distribution depended on leaf size that larger leaves invest greater proportion of petiole biomass than smaller leaves, which collectively indicates a proportional reduction in lamina area compared to the investment in leaf dry biomass. The LMA increases in resource-poor, light-intense, high-altitude or arid habitats (Hanley et al., 2007). However, the increase in LMA caused by different habitats are extremely different (Wright et al., 2002). Because LMA is a composite parameter, the effect of light intensity on LMA is a synergistic effect of leaf density and thickness (Onoda et al., 2008). LMA increases with increasing light radiation and this phenotypic plasticity show differences between species (Niinemets et al., 1998; Valladares et al., 2000). This is consistent with Liu et al. (2010), which they showed that LMA were significant differences between the two light levels even for leaves of the same individual plant. While the maximum photosynthetic rate of *Symplocos setchuensis* differed significantly between the two light level*s*, there was no significant difference in LMA between the two, suggesting that species specificity also affects the expression of plasticity in LMA. This variation is ecologically significant, as plants in low light habitats capture light energy better by increasing leaf spread per unit mass, while in high light habitats they make better use of light energy by increasing dry matter content per unit area (Poorter et al., 2009). We speculate that the foliage leaf area of bamboos distributed at middle altitude is smaller and less shading to light, which is more favorable to the growth of ferns. Conversely, tall trees with dense vegetation and large canopy cover are mainly distributed at high altitude. Due to the shading of trees, the fronds of ferns are exposed to less sunlight, leading to a reduction in the LMA. This finding is consistent with the results of Zhu et al. (2011) on the functional traits of fern fronds under 16 different light habitat conditions. Ferns confined to two habitat conditions, the natural forest shade understory and disturbed open habitats adopted different cost effective strategies for fern fronds due to differences in light conditions. This phenomenonlogy reflects a response to an increase in the availability of light owing to the low foliage density of the bamboo community typical at the mid-elevation of the study site.

## 6 Conclusion

The results of this study show that, at the individual plant and species level, the scaling relationship for fern leaf area vs. dry biomass follows the diminishing returns hypothesis, but that the numerical values of the scaling exponents differ among three elevations in the study region. We speculate that the differences in the numerical values of the scaling exponents reflect differences in the ambient light variability. Collectively, our data indicate that the leaf area vs. biomass scaling relationship of ferns is consistent with prior studies focusing on seed plant species (angiosperms and gymnosperms) showing that leaf biomass investments are responsive to environmental factors, such as the availability of light.

## Data availability statement

The raw data supporting the conclusions of this article will be made available by the authors, without undue reservation.

## Author contributions

SC, DC, QZ and DH conceived the ideas and designed methodology; SC, JL, and JS collected the data. SC and JL analyzed the data; SC, JS, DH and DC led the writing of the manuscript. All authors contributed to the article and approved the submitted version.
