# Supplementary figures and images for: “Diminishing returns” and leaf area-biomass scaling of ferns in subtropical ecosystems
Source: Front Plant Sci. 2023 Jun 27;14:1187704. doi: 10.3389/fpls.2023.1187704 (PMC10333482; doi:10.3389/fpls.2023.1187704)

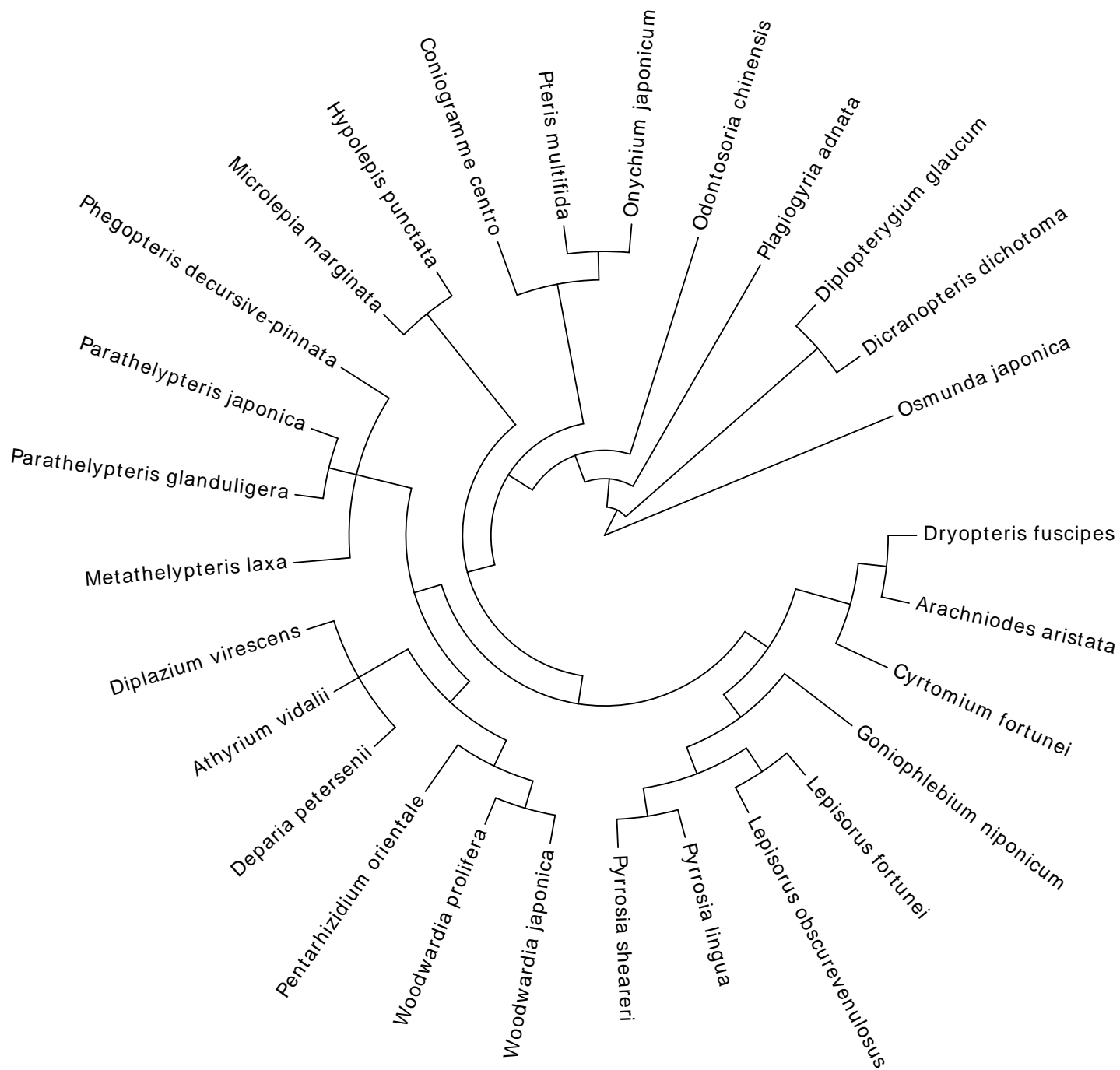

Supplement: Supplementary file 1 [file DataSheet_1.pdf]
